# Nanomaterials for Energy Harvesting

**DOI:** 10.3390/nano13071154

**Published:** 2023-03-24

**Authors:** Daniela Dragoman

**Affiliations:** 1 Physics Faculty, University of Bucharest, 077125 Măgurele, Romania; daniela@solid.fizica.unibuc.ro; 2 Academy of Romanian Scientists, 050044 Bucharest, Romania

Energy harvesting is no longer simply an academic issue; it has grown into a problem with real industrial and even social significance. We should harvest energy not only because it is plentiful and most of it renewable, but also because it could protect the environment from further pollution and, as a result, maintain the health of the population. There is still a long way to go until we reach this goal, but significant progress has been made in research of late, especially using nanomaterials. Nanomaterials and nanostructures have enhanced the performance of many energy-harvesting devices due to the increase in strength of physical effects, such as photovoltaics, piezo-, ferro-, or thermo-electricity. This is because, at this scale, interfaces and surfaces play an increasing role in light and phonon scattering, while quantum effects such as tunneling and ballistic charge carrier transport often inspire new architectures for energy harvesting and demand nanomaterials and nanostructures with precisely controlled properties. The Special Issue *Nanomaterials for Energy Harvesting* strives to showcase specially designed nanosystems as well as specific configurations and devices that could efficiently harvest energy in a certain wavelength range, and includes contributions related to their theoretical modeling, fabrication, and characterization.

Several energy sources can be harvested, such as electromagnetic energy directly from the Sun or from human activities (for instance, from wireless 5G or 6G communication networks), or environmental waste heat, among others. As such, energy harvesting can be achieved via numerous physical principles, and specific devices are designed to work efficiently in certain frequency bands.

In the ultraviolet, visible, and near-infrared spectral ranges, light conversion into electricity is mostly performed using solar cells. Research in this domain has grown steadily from the discovery of the first practical silicon solar cell [1] and has focused on finding suitable and cheaper materials, as well as novel configurations, that are able to harvest solar energy in a wider energy range with an efficiency that overcomes the Shockley–Queisser limit imposed for a single p-n junction [2]. This Special Issue contains two articles that address the problem of the performance enhancement of solar cells. The first [3] proposes a highly efficient solar cell based on crystalline silicon deposited on a back reflector Al grating covered with graphene, with a textured TiO_2_ layer on top of the active silicon and covered with metallic plasmonic nanoparticles. This solar cell is intended to work in the visible and near-infrared regions, with the proposed configuration incorporating designer techniques to increase its efficiency. Extensive numerical simulations show the influence of relevant geometric parameters and plasmonic metals on the performance of the solar cell, optimizing the structure. The second article [4] focuses on a different type of solar cell inspired by the natural photosynthesis process, namely the dye-sensitized solar cell, and investigates the ability of TiO_2_ nanoparticles and their composite with core-shell structures to enhance its efficiency by optimizing the configuration of a TiO_2_ photoelectrode. The aim is to show that a double-barrier configuration consisting of a bottom layer of TiO_2_ nanoparticles and a top layer of composite nanoparticles and SiO_2_-TiO_2_ core-shell structures deposited on a photoelectrode film increases the number of adsorbed dye molecules, and hence light absorption, as well as enhancing light scattering and decreasing the recombination rate. The net result is an increase in the optimized fabricated dye-sensitized solar cells.

On the contrary, the most common energy harvester beyond the near-infrared spectral region, e.g., beyond 2 μm and up to 100 μm, which corresponds to frequencies beyond 3 THz, is the so-called rectenna. It consists of a receiving antenna integrated with a rectifying diode. Rectennas also function as complementary devices to photovoltaic cells in the visible and near-infrared spectral regions, being able to harvest the Earth-emitted energy even at night, but are indispensable at higher wavelengths/lower frequencies. In particular, beyond 5 THz, the common Schottky diode rectifiers cannot operate, and other high-frequency rectifiers are necessary. These include devices based on quantum tunneling, ballistic or quasi-ballistic transport, exclusively based on nanostructures and/or nanomaterials. The review [5] in this Special Issue offers an overview of the specific issues, state-of-the-art performances defined with respect to a set of well-established criteria, and future research directions in THz rectifier technology. The authors discuss in detail the vertical metal–insulator–metal, as well as the metal–multi insulator–metal diode configurations, also offering insights into the planar, asymmetric geometrical diode configuration. The latter is in turn investigated in two articles, from both a theoretical as well as an experimental point of view. More precisely, in [6], parameters such as the nonlinearity, asymmetry, or responsivity of the inverse-arrowhead-shaped graphene geometric diode are explored for different neck widths and are shown to improve as the neck width decreases. The study is performed for CVD-grown graphene, which is prone to mass-production, and is supported by particle-in-cell Monte Carlo simulations. The second article [7] extends the previous numerical investigation to a new planar configuration, the so-called Z-shaped geometric diode, and investigates both ballistic and quasi-ballistic transport regimes of charge carriers—regimes that are sensitive to the device geometry. By comparing the two configurations: inverse-arrowhead and Z-shaped, it was shown that the asymmetry of the latter, which is an essential parameter of a rectifying diode, is significantly higher than that of the former, and that this parameter reaches a maximum value for a certain mean free path of charge carriers. This result is caused by the difference in geometry of the considered configurations, which leads to differences in quantum wavefunction transmission, particularly at reverse biases.

In fact, graphene is only one nanomaterial among others, such as oxides with few nanometer thicknesses, atomically-thin molybdenum disulfide layers, and carbon nanotubes that, due to their physical properties, are part of the most innovative advances for ambient electromagnetic energy harvesting. This issue is reviewed in [8], which emphasizes the advantages of using nanomaterials and nanostructures of different types in electromagnetic harvesting, using rectennas based on tiny Schottky and metal–insulator–metal diodes, two-dimensional materials such as graphene and MoS_2_, HfO_2_-based ferroelectrics, and carbon nanotubes. The challenges and perspectives of this research domain are also discussed in this review.

Aside from harvesting electromagnetic energy, the transformation of lost heat into electricity is also of fundamental importance. Achieving this goal requires stable and efficient thermoelectric materials, which should not contain toxic, rare, or expensive elements. A study on such a topic can be found in [9], where β-FeSi_2_ and its alloy with Co, β-Fe_0.95_Co_0.05_Si_2_, were identified as suitable materials. The overall performance of a thermoelectric material is quantified by the dimensionless figure of merit ZT, which depends on both the thermal and electrical conductivities of the material, as well as on the Seebeck coefficient. For the first material, the figure of merit was shown to increase by nanostructuring, i.e., decreasing the crystallite size up to tens of nanometers, at high temperatures; whereas for the alloy, despite the small amount of added Co, better performances were observed for the whole temperature interval.

Once harvested, energy should be stored. Batteries are essential devices for performing this task. In particular, lithium-ion batteries with silicon-based anodes are investigated in [10]. Whereas crystalline silicon forms silicon–lithium compounds leading to an initial coulombic efficiency of around or less than 80%, and hence a loss of lithium of about 20% during the first discharge of the anode, this parameter increases up to 90% if nanometer-scale silicon flakes are used, mixed with conductivity enhancement additives such as Super P. At the same time, high-capacity retention after long cycling is assured due to the tight binding of the porous graphitic carbon structure (formed after anode pyrolysis) with individual silicon flakes, which protects silicon against excessive irreversible reactions with the electrolyte.

In summary, the articles in this Special Issue emphasize that nanomaterials (two-dimensional materials, nanoparticles, nanotubes) and nanostructures are essential for increasing the performance of energy harvesting devices, irrespective of the energy source (electromagnetic radiation or heat), due not only to their properties and specific physical phenomena at the nanoscale, but also to the novel device configurations inspired by them. Energy harvesting, which is an increasing area of research, as well as energy storage, could thus benefit from such nanomaterials and nanodevices.

## References

[1] Chapin D.M., Fuller C.S., Pearson G.L. (1954). A new silicon p-n junction photocell for converting solar radiation into electrical power. J. Appl. Phys..

[2] Shockley W., Queisser H.J. (1961). Detailed balance limit of efficiency of p-n junction solar cells. J. Appl. Phys..

[3] Elrashidi A., Elleithy K. (2022). High-efficiency crystalline silicon-based solar cells using textured TiO_2_ layer and plasmonic nanoparticles. Nanomaterials.

[4] Zaine S.N.A., Mohamed N.M., Khatani M., Shadid M.U. (2022). Nanoparticle/core-shell composite structures with superior optical and electrochemical properties in a dye-sensitized solar cell. Nanomaterials.

[5] Citroni R., Di Paolo F., Livreri P. (2022). Progress in THz rectifier technology: Research and perspectives. Nanomaterials.

[6] Wang H., Jayaswal G., Deokar G., Stearns J., Costa P.M.F.J., Moddel G., Shamim A. (2021). CVD-grown monolayer graphene-based geometric diode for THz rectennas. Nanomaterials.

[7] Stearns J., Moddel G. (2021). Simulation of Z-shaped graphene geometric diodes using particle-in-cell Monte Carlo method in the quasi-ballistic regime. Nanomaterials.

[8] Dragoman M., Aldrigo M., Dinescu A., Vasilache D., Iordanescu S., Dragoman D. (2023). Nanomaterials and devices for harvesting ambient electromagnetic waves. Nanomaterials.

[9] Abbassi L., Mesguich D., Berthebaud D., Le Tonquesse S., Srinivasan B., Mori T., Coulomb L., Chevallier G., Estournès C., Flahaut E. (2021). Effect of nanostructuring on the thermoelectric properties of β-FeSi_2_. Nanomaterials.

[10] Tzeng Y., Jhan C.-Y., Wu Y.-C., Chen G.-Y., Chiu K.-M., Guu S.Y.-E. (2022). High-ICE and high-capacity retention silicon-based anode for lithium-ion battery. Nanomaterials.

